# ECG QT-I nterval Measurement Using Wavelet Transformation

**DOI:** 10.3390/s20164578

**Published:** 2020-08-15

**Authors:** Takao Ohmuta, Kazuyuki Mitsui, Nitaro Shibata

**Affiliations:** 1 Department of Clinical Engineering, Faculty of Medical Engineering, Suzuka University of Medical Science, Mie 510-0293, Japan; 2 Department of Advanced Machinery Engineering, School of Engineering, Tokyo Denki University, Tokyo 120-8551, Japan; mitsui@cck.dendai.ac.jp; 3 Shinjuku Mitsui Building Clinic, Tokyo 163-0404, Japan; shibata@isk-smbc.org

**Keywords:** wavelet transform, ECG recognition, QT interval

## Abstract

Wavelet transformation, with its markedly high time resolution, is an optimal technique for the analysis of non-stationary waveform signals, such as physiological signals. Therefore, wavelet transformation is widely applied to electrocardiographic (ECG) signal processing. However, an appropriate application method for automated QT-interval measurement has yet to be established. In this study, we developed an ECG recognition technique using wavelet transformation and assessed its efficacy and functionality. The results revealed that the difference between the values obtained using our algorithm and the visually measured QT interval was as low as 4.8 ms. Our technique achieves precise automated QT-interval measurement, as well as Te recognition, that is difficult to accomplish even by visual examination under the electromyography noise environment.

## 1. Introduction

The electrocardiographic (ECG) QT interval indicates the action potential duration (and refractory period) of the ventricular muscle [1]. The QT interval can be used for evaluation of the effect of antiarrhythmics, classification, and severity of QT prolongation syndrome and as an indicator for electrophysiological instability of the ventricle [2]. The QT interval is directly affected by the autonomic nervous system, particularly the sympathetic nervous system, and indirectly affected via the heartbeat interval. Therefore, the condition of the autonomic nervous system can be estimated based on changes in the QT interval [3].

The beginning of the wave and the end of the T wave must be precisely recognized to determine the accurate QT interval. The beginning of the QRS wave can be easily determined, whereas the end of the T wave (Te) is often difficult to identify because the wave is relatively shallow. Lepeschkin et al. identified the end of T wave on the basis of T and U wave shapes by classifying the polarities of T and U waves into the following four types, for a total of 16 patterns: (1) positive, (2) positive former and negative latter halves, (3) negative former and positive latter halves, and (4) negative [4]. Following this method, the intersection of the baseline with the tangent to the descending portion of the T wave is defined as Te.

Conventionally, QT intervals are measured manually using calipers on an extended electrocardiogram or via computer automation. Using the manual method, it is difficult to achieve a measurement resolution of millisecond-order accuracy because of the ECG recording speed and/or the T-wave shape and both intra- and inter-observer measurement differences are common. In the case of the computer automation method, the development of a valid measurement algorithm is required to establish a highly precise automated measurement technique.

Fujiki et al. used a simple algorithm to measure the duration between the beginning of the Q wave and the apex of the T-wave (aT) interval via Holter ECG, instead of QT end interval [5]. This measurement method provides precise and prompt recognition [6]; however, it does not measure the “real” QT interval.

Many automatic QT measurement techniques have been developed, including the threshold-based method, the tangent based method, the fitting model method, and the support vector machines method [7]. However, the accuracy of these methods is not satisfactory under the noisy recording conditions caused by electrical interference (AC), movement of chest cavity by respiration, peripheral muscle myogram, and/or atrial arrhythmias such as atrial flutter [8].

The main advantage of wavelet transformation is the markedly higher time resolution compared with conventional waveform signal analysis methods such as fast Fourier transformation. Therefore, wavelet transformation may provide an improved technique for the analysis of nonstationary waveform signals. Wavelet analysis has become a renowned tool for characterizing ECG signals, and efficient algorithms have been reported for multilead ECG detection [9]. Additionally, recognition by single-lead ECG [10,11] has been reported. Wavelet transformation represents a potential alternative to empirical methods because it is an evidence-based measurement technique that can detect characteristic waveforms in ECG noise removal or reduction, the P wave [12], QT interval, ST interval, and equipotential level of the baseline.

In this study we have developed an ECG waveform recognition technique using wavelet transformation and assessed its usefulness in a single induction with a sampling frequency of 1 kHz.

## 2. Subjects and Methods

### 2.1. Subjects

We studied 17 subjects (age range: 21 to 72, mean 39 ± 20.1 years, 12 males) who underwent continuous electrocardiography as an orthostatic examination. We obtained continuous digital recordings using a computer connected to a lead II ECG with a multi-telemeter system (WEB-5000, Nihon Kohden, Tokyo, Japan) at a sampling frequency of 1 kHz.

Informed consent was obtained from all participants. All experimental procedures were approved by the Human Life Ethics Committee of Tokyo Denki University (27–46). This study was conducted in accordance with the Helsinki Declaration as revised in 2013.

### 2.2. ECG Recognition Using Wavelet Transformation

When a signal with noise is *x*(*t*), the control template signal is *g*(*t*), and the position of the template signal on the time axis is τ, the occurrence time point and magnitude of the signal can be determined by convolution as shown in Equation (1) with the superposition of *g*(*t*) on *x*(*t*) while shifting it in the time axis direction:

(1) $$y\left(t\right)=\int_{-\infty }^{\infty }g^{*}\left(t-\tau \right)x\left(t\right)dt.$$

Since the waveform *g*(*t*) of each component of *x*(*t*) is unknown, arbitrary waveforms (mother wavelet) are captured (wavelet function system) and compared with *x*(*t*), which constitutes the wavelet transformation.

The wavelet transformation *W*(τ,2*^j^*), with the mother wavelet ψ(*t*) function *x*(*t*), can be expressed as Equation (2), where *j* is a scale parameter:

(2) $$W\left(\tau ,2^{j}\right)=\frac{1}{\sqrt{2^{j}}}\int_{-\infty }^{\infty }\psi \left(\frac{t-\tau }{2^{j}}\right)x\left(t\right)dt.$$

Although there are various methods for scale transformation of a mother wavelet to obtain the wavelet function system, in our ECG recognition technique, we use 2*^j^* on the basis of the scale parameter *j* (discrete wavelet transformation). In this case, *j* represents a high- or low-frequency component in the wavelet function system when the value is low or high, respectively.

For the mother wavelet ψ(*t*), we used the Mexican hat, that is, the second derivative of the Gauss function, which is a well-known wavelet function that can better detect waveform peaks and inflection points, as shown in Equation (3):

(3) $$\psi \left(t\right)=\left(1-2t^{2}\right)e^{-t^{2}}.$$

Indices with a high correlation were extracted through pattern matching of wavelet functions *j* = −2 to 2 after scale transformation with ECG waveforms. The ECG waveform recognition points (R, aT, and Te) were determined by calculating *t* (*t*_−2_ through *t*_2_ indicate the times of the indices) using Equation (4) according to the indices in a box. Equation (4) was used because if the QRS and T-wave are asymmetrical, the simple averaging method will produce worse recognition results:

(4) $$t=\frac{\frac{\frac{\frac{t_{-2}+t_{-1}}{2}+t_{0}}{2}+t_{1}}{2}+t_{2}}{2}.$$

**Figure 1:**
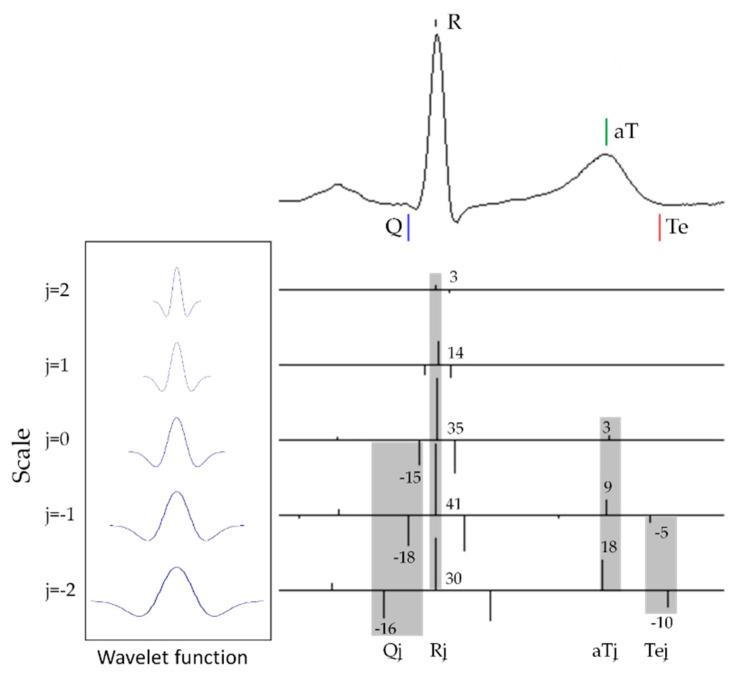
Example of a wavelet transformation of ECG.

Figure 1 illustrates an ECG waveform of the wavelet function system as the respective converted waveforms of the mother wavelet with scale parameter *j* = −2~2 and its features (Q*_j_*, R*_j_*, aT*_j_*, and Te*_j_*, as gray areas on the scale below each part of the ECG) extracted through wavelet transformation processing. These features were determined by sampling the maximum values (lines in the positive direction) and minimum values (lines in the negative direction) obtained through wavelet transformation processing using the wavelet function system and ECG waveform, as shown in the left panel of Figure 1.

**Figure 2:**
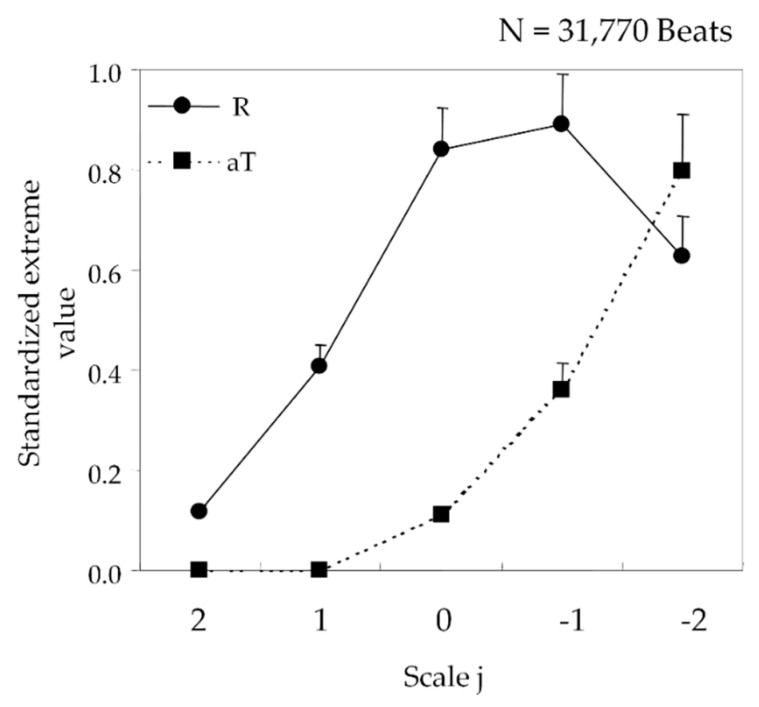
Changes in the features of R and aT for 12 subjects.

Although individual differences in ECG waveforms can be revealed, it is not possible to recognize each of the parts based on the extreme values obtained. Therefore, recognition of the individual parts of an ECG waveform was performed by analyzing the changes in the extreme values (Figure 2).

In an ECG waveform, the apexes of the QRS (R) and T waves (aT) correspond to the maximum values of the features extracted through wavelet transformation processing. To discriminate between the two, we investigated the differences in change patterns in the features. Looking at R_0~2_ and aT_0~2_, the features of R and aT, respectively, in Figure 1, aT increases with the values on scale *j* (aT_0_ < aT_1_ < aT_2_), whereas R_j_ reaches a maximum at R_1_ (R_1_ > R_2_, R_1_ > R_0_). This feature was observed in the ECG shown in Figure 1 and in 31,770 beats of 12 subjects aged 20–49 years (Figure 2). After the maximum values of the QRS and T waves are discriminated, the Q and T-wave ends are determined as the minimum value in the time interval earlier than R and the minimum value in the time interval later than aT, respectively.

## 3. Validation of ECG Recognition Efficacy

The following two tests were conducted to validate the efficacy of our ECG recognition technique.

### 3.1. Noise Tolerance Simulation

We synthesized various potential noises in the ECG waveform (normal ECG) during ECG measurement of a total of 2669 heartbeats to test the validity of our ECG recognition technique. The following types of noise were assessed:

(a) commercial power noise: 0.2 mV, 50/60 Hz sine wave
(b) ambulatory noise: 0.5 mV, 2 Hz sine wave
(c) respiratory noise: 0.5 mV, 0.25 Hz sine wave
(d) atrial flutter noise: 0.4 mV, 5 Hz sawtooth wave
(e) electromyographic (EMG) noise: white noise and 1 mV, 20–40 Hz sine wave.

### 3.2. Validation of Recognition Points

Recognition of ECG Q, R, aT, and Te was conducted using our ECG recognition technique and visual examination to determine the QT interval and differences between these (absolute) values.

Visual ECG examination was conducted by three specialists in ECG measurement, and the resulting values were averaged. For each subject, the standing ECG of 10 consecutive heartbeats was recorded every 3 min for 30 min to determine the QT intervals of 60–100 beats.

## 4. Results

### 4.1. Results of Noise Tolerance Simulation

**Figure 3:**
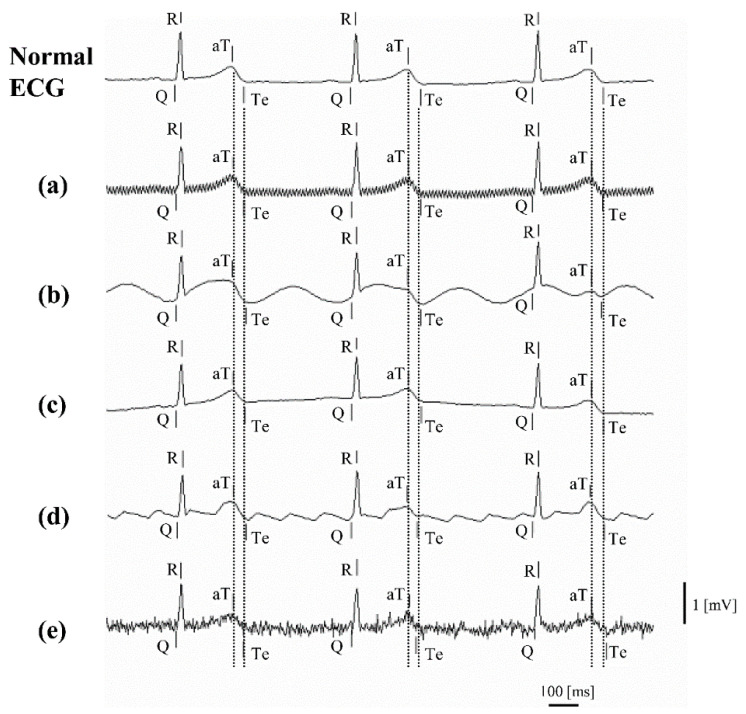
Noise tolerance simulation.On a normal ECG, (**a**) commercial power noise (50 Hz), (**b**) ambulatory noise, (**c**) respiratory noise, (**d**) atrial flutter noise, and (**e**) EMG noise were synthesized to test our ECG recognition technique. In this figure, the vertical lines beside the letters Q, R, aT, and Te indicate recognition points.

The results of the noise tolerance simulation and the differences in recognition points between the normal ECG and the ECG containing various types of noise are shown in Figure 3 and Table 1, respectively. Under noise conditions (a) to (c), our ECG recognition technique recognized Q, R, aT, and Te at almost the same points as on the normal ECG (error range: −0.2 ± 2.7 ms). Under noise condition (e), EMG noise, the recognition error range was ±8 ms even for Te, which was recognized visually with difficulty. Under noise conditions (a), (b), (c), and (e), the QT interval was well recognized by automated recognition. However, under noise condition (d), the error range was as large as ±15 ms. Synthesized sawtooth wave peaks between aT and Te form a pseudo two-phase T wave (with two T wave apexes), leading to the measurement of longer QT intervals than the actual values, with a mean of 9.4 ms (Table 1).

### 4.2. Validity of Recognition Points

**Figure 4:**
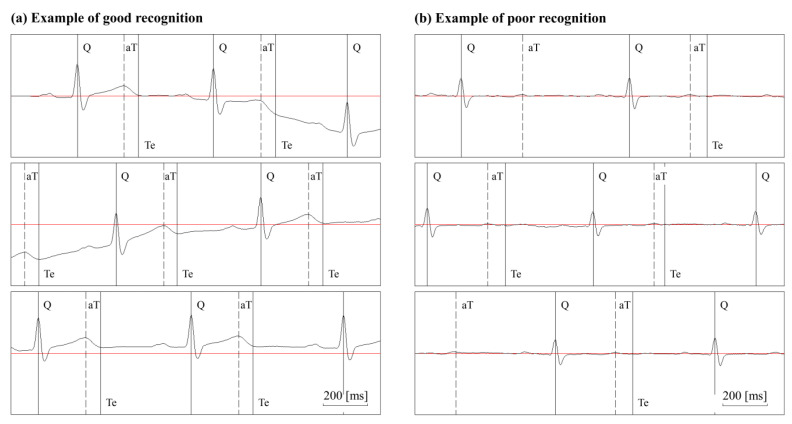
Examples of automated ECG recognition measurement. The Q wave, apex of the R wave, apex of the T wave (aT), and T-wave end (Te) were recognized on consecutive electrocardiograms. (**a**) Even in the presence of baseline drifting, aT and Te are precisely recognized. (**b**) A flattened T wave makes recognition of aT and Te difficult.

Examples of good and poor recognition are illustrated in Figure 4. Even in the presence of drifting due to respiration and motion, aT and Te were precisely recognized (Figure 4a). Recognition of aT and Te was difficult in the flattened T wave (Figure 4b); however, recognition is difficult even using visual examination in such a case; therefore, it is generally excluded from the measurement.

The number of visually counted heartbeats was 1610. The variance of the manually annotated results, annotated by the experts, was 11.2 ms. The difference between our ECG recognition technique and the visual measurement was 4.8 ± 2.6 ms (Table 2).

## 5. Discussion

Previously developed T-wave end-recognition methods include the differential, threshold, slope, least-square, and T-wave area methods. The presence of certain fluctuations of the baseline, as well as EMG and atrial flutter/fibrillation, makes analysis difficult, as the algorithm becomes complicated when considering these factors. However, even with various types of noise, our ECG recognition technique using wavelet transformation successfully recognizes Te, which has been an issue with other methods, and produces a difference in measurement values as low as 4.8 ms when compared with the values obtained by visual measurement. These results demonstrate that our ECG recognition technique achieves an accurate automated QT-interval measurement.

One of the limitations of our ECG recognition technique is that some shapes of T wave may be difficult to measure. In this study, because the true QT interval cannot be detected from a waveform containing actual noise, we conducted our evaluation via simulation. The simulation using condition (d), atrial flutter noise, in Figure 2, indicates that our ECG recognition technique cannot be applied to abnormal T waves including the two-phase T wave and negative T wave (T wave with a convex lower shape) because of the maximum value used in our algorithm for recognition of the T wave. Further investigation is required in this area.

## 6. Conclusions

In this study, we evaluated the efficacy of our ECG recognition technique using wavelet transformation. The tolerable error in a QT-interval measurement is approximately 20 ms for clinical use and 5 ms for ICH E14 QT/QTc evaluation [13]. Our algorithm produces a difference in QT-interval measurements as low as 4.8 ms from the values obtained by visual measurement. This novel method achieves a highly precise automated QT-interval measurement and Te recognition, which is difficult to recognize visually under an EMG noise environment.

## Figures and Tables

**Table 1:** Differences in recognition points between normal ECG and ECG containing various types of noise.

Type of Noise	Q	R	aT	Te	QTe
(a) Commercial power noise 50 Hz	−0.5	−0.6	−0.9	0.4	1.0
Commercial power noise 60 Hz	−0.5	−2.3	0.1	0.0	0.5
(b) Respiratory noise	0.0	0.0	0.0	0.0	0.0
(c) EMG noise	−3.6	−2.2	7.4	0.0	3.6
(d) Atrial flutter noise	−5.8	0.0	11.3	3.5	9.4
(e) Ambulatory noise	0.1	0.1	−0.2	1.1	1.0

Measurement unit (ms).

**Table 2:** Difference in QT-interval measurement between our ECG recognition technique and visual examination.

Subjects	Heartbeats Used for Comparison	Difference from Visual Evaluation (ms)
(1) 21 M	100	3.8
(2) 22 M	100	2.1
(3) 22 M	100	2.0
(4) 22 M	100	3.9
(5) 22 M	100	4.2
(6) 22 M	100	7.8
(7) 22 M	70	5.8
(8) 23 M	100	12.3
(9) 32 M	100	5.3
(10) 55 M	100	4.3
(11) 56 M	100	4.0
(12) 72 M	80	4.5
(13) 25 F	60	3.8
(14) 49 F	100	3.0
(15) 61 F	100	3.8
(16) 65 F	100	8.5
(17) 71 F	100	2.0
-	1610	4.8 ± 2.6

Numbers outside parentheses show subjects’ ages. M: male, F: female.
